# Editorial: Exercise priming: The use of physical exercise to support motor and cognitive function

**DOI:** 10.3389/fpsyg.2022.1043611

**Published:** 2022-10-10

**Authors:** Anjali Sivaramakrishnan, Micah Zuhl, Cameron S. Mang

**Affiliations:** ^1^Department of Physical Therapy, School of Health Professions, UT Health San Antonio, San Antonio, TX, United States; ^2^School of Health Sciences, Central Michigan University, Mount Pleasant, MI, United States; ^3^Faculty of Kinesiology and Health Studies, University of Regina, Regina, SK, Canada

**Keywords:** cognition, motor skill acquisition, exercise, cognitive performance assessment, priming

## Introduction

Exercise priming is a novel concept that utilizes the neural stimulating effects of physical exercise to assist with learning, memory, and skill retention of a task. In clinical practice, a bout of exercise performed in close proximity (either before or after) to behavioral treatment may improve an individual's ability to retain the information learned in therapy. For example, the partnership of exercise with treatment for depression and anxiety has been shown to be more effective at improving symptoms than treatment alone (Lee et al., 2021). Exercise priming has also been explored in combination with motor skill training. This is demonstrated by improved motor skill retention among stroke patients who performed a brief bout of aerobic exercise prior to physical therapy (Nepveu et al., 2017). Even further, exercise priming has been proposed as a novel approach to improve skill development for athletes (Statton et al., 2015).

The mechanisms that explain exercise priming are complex and remain to be fully elucidated. One proposed explanation is that aerobic exercise increases brain blood flow and oxygenation, which may promote higher cortical activation in prefrontal regions of the brain that are responsible for working memory, attention, and executive function. Another possible mechanism is enhanced neural plasticity via exercise-induced upregulation of neurotrophic factors such as brain-derived neurotrophic factor (BDNF) and vascular endothelial growth factor (VEGF). In addition, neuroendocrine responses to exercise may help to explain the exercise priming effect. Specifically, catecholamines released during short duration bouts of exercise have been shown to promote arousal and attention. Evidence also suggests that exercise-induced release of cortisol via activation of the hypothalamic-pituitary-adrenal (HPA) axis improves learning and memory. In this Research Topic, we invited scientists to further explain and comment on the mechanisms of exercise priming along with analyzing the potential benefits of exercise priming in learning scenarios. We have summarized and discussed the major findings and application aspect of each article below.

## Summary of findings

Cognitive control includes the ability to monitor actions and suppress those that may be undesirable. Wunder and Staines used event-related potentials (ERPs) extracted from electroencephalography to compare cortical activity associated with “response conflict” in individuals reporting high and low levels of physical activity. ERPs linked to conflict monitoring, error processing, and attentional resource allocation were examined during performance of a modified flanker task with varying degrees of response conflict. Notably, highly active participants demonstrated modulation of performance and ERPs corresponding to the degree of conflict provided in flanker task trials. Similar modulations based on the degree of response conflict were not observed in participants reporting low physical activity levels. The authors suggest that highly active individuals may demonstrate greater adaptability of cognitive control compared to those performing low levels of physical activity. Future work may consider whether and how habitual physical activity affects cortical activity that supports cognitive function.

As previously discussed, exercise priming is the partnership between exercise and performance on a cognitive demanding task. Moriarty et al. investigated the effect of either acute moderate or high intensity aerobic exercise on piano performance among university music majors; and, further aimed to determine if performance was linked to exercise-induced activation changes within the primary motor cortex (M1) using functional near infrared spectroscopy (fNIRS) techniques. Piano performance scores were higher after 15 min of moderate intensity cycling exercise compared to a sedentary control trial, but not after 15 min of intense exercise. They further reported a significant linear relationship between post-exercise M1 activation and piano performance, which highlights neural activation as a possible mechanism explaining the exercise priming effect. The authors note that moderate intensity may be a more effective approach if the goal of exercise priming is to improve performance of a motor skill activity. This was further demonstrated by the report of a negative relationship between exercise heart rate during intense exercise and piano performance outcomes. The creative work of Moriarty et al. demonstrates that brief moderate intensity exercise may be more suitable for improving motor skill tasks. Limitations include a small sample size and not controlling for exercise workloads between exercise trials. This work is a small step forward in understanding the influence of exercise on cognitive demanding tasks, and future work should continue to explore the utility of various exercise modalities and intensities on various types of cognitive tasks.

Past work indicates that high-intensity interval leg cycling increases facilitation and reduces inhibition in motor cortex circuits projecting to non-exercised muscles of the hand (Singh et al., 2014; Smith et al., 2014; Stavrinos and Coxon, 2017). These neural changes are thought to be linked to exercise-induced upregulation of neurotrophic factors (Knaepen et al., 2010; Mang et al., 2013). The majority of studies on this topic have included adults with high physical activity levels and fitness or have not reported on physical activity and fitness status (Mellow et al., 2020). Findings presented by Hendy et al. extend prior research to the sedentary population. Using a randomized cross-over study design, transcranial magnetic stimulation was delivered and venous blood samples collected from sedentary young adults before and after a 20 min bout of high-intensity interval training (HIIT) on a stationary leg cycle or a period of rest. HIIT increased corticospinal excitability and decreased motor cortical inhibition, similar to past reports, but did not change systemic concentration of neurotrophic factors. The authors speculate that the cortical changes may support motor outcomes if HIIT were paired with motor practice. A priority for future research will be to translate such work into real-world motor learning applications.

Pickersgill et al. reviewed the intersections of exercise, diet, and sleep patterns on neuroplasticity. They discuss the neurophysiological mechanisms underlying exercise priming and suggest that a combination of aerobic and resistance exercise may be superior to either form of exercise alone for upregulating biomarkers of neuroplasticity (Marinus et al., 2019). Dietary factors are an important aspect to consider, and the authors suggest that a ketogenic, Mediterranean, gluten free diet, supplemented with curcumin and omega-3 fatty acids could improve cognition and other neuroplasticity measures (McCann and Ames, 2005; Petersson and Philippou, 2016). Sleep deprivation can negatively affect corticospinal excitability, reduce intracortical inhibition and prolong the cortical silent period (Scalise et al., 2006). Overall, there is an interplay between all three factors and regular exercise coupled with a healthy diet and adequate sleep is necessary for maintaining good brain health.

## Author contributions

Manuscript conceptualization, original draft preparations, and revisions were completed by AS, MZ, and CM. All authors contributed to the article and approved the submitted version.

## Conflict of interest

The authors declare that the research was conducted in the absence of any commercial or financial relationships that could be construed as a potential conflict of interest.

## Publisher's note

All claims expressed in this article are solely those of the authors and do not necessarily represent those of their affiliated organizations, or those of the publisher, the editors and the reviewers. Any product that may be evaluated in this article, or claim that may be made by its manufacturer, is not guaranteed or endorsed by the publisher.
